# When intents to educate can misinform: Inadvertent paltering through violations of communicative norms

**DOI:** 10.1371/journal.pone.0230360

**Published:** 2020-05-29

**Authors:** Derek Powell, Lin Bian, Ellen M. Markman

**Affiliations:** 1 Department of Psychology, Stanford University, Stanford, CA, United States of America; 2 Department of Human Development, Cornell University, Ithaca, NY, United States of America; Pacific Lutheran University, UNITED STATES

## Abstract

Paltering is a form of deception whereby true statements are used to mislead and is widely employed in negotiations, marketing, espionage, and ordinary communications where speakers hold ulterior motives. We argue that paltering is accomplished through strategic violations of communicative norms such as the Gricean cooperative principles of relevance, quantity, quality and manner. We further argue that, just as genuine paltering deceives by deliberately violating communicative norms, inadvertent violations of these norms may be just as misleading. In this work, we demonstrated that educational information presented prominently on the American Diabetes Association website violated the Gricean communicative principles and disrupted readers’ performance on a test of diabetes knowledge. To establish the effects of these communicative violations, we revised the ADA's information to preserve the original content while better adhering to pragmatic principles. When these ADA explanations were judiciously revised to minimize pragmatic violations, they were transformed from misleading to educational.

## Introduction

Telling the truth can be used to mislead. The act of deceiving through truthful statements is known as “paltering” [1] and is widely employed in negotiations (e.g., [2]), political discourse (e.g., [3,4]), marketing and sales (e.g., [5,6]), and espionage (e.g., [7]) as well as in many ordinary interactions where speakers hold ulterior motives (e.g., [8,9]). Palterers imply rather than assert falsehoods, leading listeners to draw false inferences from true statements. Paltering is sometimes preferable to flat-out lying because hewing closer to the truth can be more persuasive, can be less likely to be discovered, if discovered can preserve deniability, and can hide speakers’ bias (e.g., [10]). Paltering also offers a way to deceive without experiencing the guilt of lying outright [2]. Although genuine paltering is a form of deliberate deception, inadvertent paltering is also possible. What would count as inadvertent paltering? Following Malle and Knobe’s [11] model of a folk concept of intentionality, we suggest that inadvertent paltering takes place when the speaker (a) does not desire to deceive and (b) is not aware of communicating in a misleading way. We have previously presented one case-study in which well-intentioned speakers inadvertently engaged in paltering ([12]; also see [10] for further instances of misleading through pragmatic implications). In the current work, we argue that deliberate paltering is accomplished through strategic violations of communicative norms and that inadvertent violations of these same norms can be equally misleading. We demonstrate the consequences of inadvertent paltering in materials presented on a widely-respected health website.

We first turn to one of the foundational theories of pragmatics formulated by Grice [13]. One of the fundamental insights of linguistic pragmatics is that listeners are continually drawing inferences, filling in blanks, interpreting, and making sense of what speakers say. It is important that these inferences are sound, reflecting the speaker’s communicative intent. Grice argued that this is made possible by speakers and listeners engaging in a cooperative enterprise where each is making assumptions about their mutual knowledge or common ground. Grice formulated a set of principles or maxims that listeners expect their communicative partners to adhere to. The assumption that a communicative partner will adhere to these principles guides listeners in their interpretations of their partners’ utterances. The Gricean principles of quality, quantity, relevance, and manner all work together to ensure successful communication.

The principle of “relevance” sets the expectation that a speaker’s utterances are relevant to the topic under discussion. This is essential for the interpretations of pronouns and determiners for example. “He, she, they, that, this” can only be correctly interpreted in a sentence if the preceding context makes it clear what they refer to. But many more subtle examples exist. For example, the utterance “It looks like rain today” as an answer to a question has radically different interpretations depending on the question asked. If asked, “Should we go on a picnic?” “It looks like rain today” means, “No, we shouldn’t”. If asked “Is the drought ever going to end?”, “It looks like rain today” means, “Yes, maybe the drought is ending.”

The principle of “quality” sets the expectation that the utterance will be truthful and that speakers have justification or evidence for believing their statements to be truthful. To illustrate, suppose you are at the airport when you overhear one passenger mention to another that their flight was delayed, and you realize this might be your flight to New York. Then, someone approaches you and asks whether the flight to New York is delayed. If you answered “yes,” the principle of quality would lead that person to assume you not only believed but had good evidence that the flight was delayed. In this case, they might decide to leave the area and go have lunch. Clearly, when evidence is merely hearsay, it is more felicitous to say, “I think so, but I’m not sure.”

The principle of “quantity” sets the expectation that speakers will provide an appropriate amount of information and detail: as much as is needed, but no more. Someone who apologizes for running late by saying “I had a hard time finding a parking space for my Porsche today,” is providing more specific information than is needed. This could lead listeners to wonder about the speaker’s communicative intent and infer the speaker is bragging as well as apologizing.

Finally, the principle of manner sets the expectation that speakers will avoid ambiguity, present information in an orderly manner, and be brief.

All of these principles can be flouted, that is blatantly violated in ways that are meant to be obvious to the listener. This technique often underlies irony, humor, metaphor, and sarcasm. Sarcasm, for example, flouts the quality principle. If someone has just mentioned seeing a really disturbing documentary on the holocaust and then says “It was a really lighthearted, upbeat movie”, listeners can readily infer she means the opposite. A listener will not be misled or deceived so long as they are able to detect the intentional violation of the principle. In contrast, deception relies on the strategic violation of these principles with the hope that listeners will not detect these violations. Outright falsehoods are clearly a violation of the maxim of quality. More subtle are the violations that constitute paltering—telling the truth but deftly manipulating other communicative principles to effectively mislead or deceive.

We turn now to some examples of genuine paltering. According to a review by Luscombe [7], palterers commonly seek to “evade certain elements of the truth through vague descriptions, misplaced emphasis and omissions” (also see [1]). We argue that these methods palterers employ to accomplish their deceptions all rely on exploiting and subtly violating the Gricean principles.

Vagueness is a violation of quantity and manner that, along with the principle of relevance, invites listeners to fill in gaps with plausible, but false, inferences. Rogers et al. [2] provide an example of an executive looking to fill a position who has identified only one candidate who fits. To reduce the leverage held by the candidate, the executive might say: “There is a great deal of demand for this position from a large number of impressive individuals.” Although it may be true that many “impressive” people applied, their credentials were not a good fit for this particular position. This deception relies on the listener interpreting the vague “impressive” as being relevant to the criteria used for hiring.

Palterers also emphasize and provide irrelevant information to deflect attention away from topics they wish to avoid (e.g., [6,9,2]). One common technique is to begin a story with details of secondary importance to the reader while postponing more essential facts [1]. Misplaced emphasis is a violation of the principle of relevance and quantity, according to which listeners are expecting a given utterance to be importantly relevant to the topic under discussion. Irrelevant information not only deflects attention, but can also be used to mislead more directly. For instance, presented with one question or topic of discussion, palterers sometimes address another. Here too, they violate the principles of relevance and quantity. Politicians are notorious for using these tactics (e.g., [3]). Emphasizing irrelevant information often goes hand-in-hand with the omission of crucial information, a further violation of the quantity principle.

Often, paltering involves violation of multiple Gricean principles. Suppose someone is selling a car and a potential buyer asks, “Does this car need any maintenance?” The car is scheduled for maintenance fairly soon, but a paltering seller might respond, “It’s a great car, it’s always run beautifully”. This violates relevance and quantity: It might be true that the car has run smoothly, but the seller is answering a different question (violating relevance) while failing to mention that the car needs maintenance soon (violating quantity).

Pragmatic violations of this sort, such as subtle and strategic implications, are widespread in marketing communications and product labeling [14]. Behavioral research has shown that pragmatic implications can be just as forceful as direct assertions in advertising contexts [15]. In some cases, a certain amount of disclosure is often legally mandated, such as in the marketing and sales of financial services and pharmaceutical products. Even in these cases, however, some companies contrive to provide just enough information to meet the disclosure requirements while still concealing many known risks from potential consumers [5], a violation of quantity. Still, if misrepresentation is significant enough, companies can be held liable for paltering communications (e.g. [16]).

Our present study focuses on how these same kinds of paltering techniques might be accomplished inadvertently if speakers do not take care to respect Gricean principles of communication. In a recent case study, Powell et al. [12] found that the American Diabetes Association’s website inadvertently paltered by presenting an infelicitous list of myths about diabetes, likely misleading health consumers. The American Diabetes Association website is a highly reputable and visible source for diabetes information [17]. This content is prominently displayed on the site and has also been linked, reproduced, or paraphrased on many other prominent health websites. In contrast to the other instances of paltering we have discussed, like Powell et al. [12], we do not believe that the ADA was trying to be misleading, but most likely meant to provide constructive information and advice.

The offending page presented a list of ten diabetes “myths” alongside ten explanations for these myths meant to correct those misconceptions [18]. Labeling a statement a “myth” marks it as both widely believed and robustly false. That is, a felicitous speaker would not label a statement a “myth” if it were false only on an uncharitable or highly technical reading. However, the ADA website listed as “myths” statements that were partly or even largely true. For instance, “Myth: People with diabetes can’t eat sweets or chocolates”. Obviously, labeling this statement a myth implies that people with diabetes *can* eat sweets and chocolates. It may even license a reader to further infer that people with diabetes may eat sweets and chocolates regularly, or just as people without diabetes do. Participants who read these infelicitous myths performed significantly worse on a test of basic diabetes knowledge than participants who had not been exposed to these infelicitous myths. Rephrasing the myths as questions which don’t have such presuppositions (e.g., “Can people with diabetes eat sweets or chocolates?”) eliminated their deleterious effects [12]. Thus, it seems that the infelicitous use of the “myth” label misled readers by an apparently unintentional form of paltering.

Powell et al. [12] focused only on the pragmatics of the ADA’s myths. As mentioned earlier, however, the ADA’s infelicitous myths were accompanied by explanations meant to clarify the myths. One striking and unpredicted finding from Powell et al. [12] was that not only did these explanations fail to counter the detrimental effects of reading the myths, the ADA’s explanations were themselves problematic. Recall that when the myths were rephrased as questions, participants’ diabetes knowledge was preserved. In contrast, when participants read the ADA’s explanations alongside those questions, their performance on the knowledge test suffered just as much as those participants who had read the infelicitous myths [12].

Here, we now argue that the ADA’s explanations were flawed because they contained the same communicative violations that characterize genuine paltering. To illustrate, consider the ADA’s explanation for the myth that “People with diabetes are more likely to get colds and other illnesses.”:

### ADA’s explanation

You are no more likely to get a cold or another illness if you have diabetes. However, people with diabetes are advised to get flu shots. This is because any illness can make diabetes more difficult to control, and people with diabetes who do get the flu are more likely than others to go on to develop serious complications.

There are a number of pragmatic violations in this explanation that have the potential to mislead readers. In the first sentence, “you are no more likely to get a cold or another illness if you have diabetes,” the use of “another illness” is vague in a way that violates the principle of manner. It’s unclear whether illnesses include only things like colds and other viral infections, or also include things like pneumonia, or even things like heart disease or kidney disease. Depending on the scope of “illness,” this statement might readily be false: for example, people with diabetes do have an increased risk of pneumonia. Another problem is that the explanation violates quantity and relevance by focusing solely on the *likelihood* of contracting an illness. When considering risk for illness, the probability of contraction is not the only relevant consideration, but also the severity of the illness and its effect on function and well-being. By not specifically clarifying that their statements pertain only to the likelihood of contraction, the ADA’s explanation falsely invites the interpretation that people with diabetes are no more affected by illnesses than people without diabetes. Delaying this explicit point and eventually focusing only on the flu is a violation of both relevance and quantity. Altogether these violations give the false impression that diabetes has little detrimental effect on the immune system.

Our goal was to examine the effects of the pragmatic violations rather than semantic content of these explanations. Thus, we sought to revise them as minimally as possible, resolving the pragmatic violations without changing the actual content being presented. As much as possible, we sought to avoid adding content that was not originally presented, but in some cases we did remove distracting content in order to correct pragmatic violations. Here is a revision crafted under these constraints that we predicted should be informative rather than misleading:

### Revised explanation

Although you may not be more likely to get a cold if you have diabetes, people with diabetes who do get the flu, for example, are more likely than others to go on to develop serious complications and any illness can make diabetes more difficult to control. For this reason, people with diabetes are advised to get flu shots.

In the revised explanation, we first addressed the violation of manner caused by the vague use of “another illness” by deleting this phrase. Then, we corrected the violations of quantity and relevance, whereby the ADA’s explanation focused solely on the likelihood of contraction. By beginning the first sentence with “although,” we explicitly set up a contrast between the likelihood of contraction and the potential severity of illness. By moving this point up in the explanation, we corrected the violation of the relevance principle. In addition, adding “for example” generalized the risk of increased severity beyond the flu by stipulating that complications from the flu were an example of a more general point.

On our analysis, five of the ADA’s explanations included violations of the four Gricean maxims, including violations of manner, relevance, quantity, or quality. Table 1 presents our analysis of the ADA’s explanations and their pragmatic violations in detail, along with the measures we took to correct them. By judiciously editing the ADA’s explanations to minimize pragmatic violations, we should be able to retain the important informational content while avoiding paltering. Once edited, the explanations should no longer mislead and instead should become informative.

**Table 1:** The ADA’s myths and accompanying explanations, along with those myths rephrased as questions, our analysis of the pragmatic violations in the ADA’s explanations, and our revised explanations. Sources supporting the factual accuracy of our revised explanations are the same as those supporting the correct and incorrect answers for the diabetes knowledge scale, referenced in Table 2.

2	**Myth:** If you are overweight or obese, you will eventually develop type 2 diabetes.		
	**Question:** If you are overweight or obese, will you eventually develop type 2 diabetes?		
	**ADA’s explanation:** Being overweight is a risk factor^1^ for developing this disease, but other risk factors such as family history, ethnicity and age also play a role.^2^ Unfortunately, too many people disregard the other risk factors for diabetes and think that weight is the only risk factor for type 2 diabetes.^3^ Most overweight people never develop type 2 diabetes, and many people with type 2 diabetes are at a normal weight^4^ or only moderately overweight.	**Revised explanation:** Being overweight does increase your risk for developing type 2 diabetes, even though other risk factors such as family history, ethnicity and age also play a role.	
	**Analysis**		
	1) Violates the maxim of manner: “Risk factor” is medical jargon; we replaced this with “increases your risk.”		
	2) Maxim of relevance suggests that these other listed factors are just as important as being overweight. Changed “but” to “even though” to reverse the implied priority of the assertions.		
	3) Violates maxims of manner and quality. Maxim of quality suggests there is a justification for saying it is unfortunate that people disregard other risk factors. However, people have no control over these factors, so it is unclear what is unfortunate about their focusing primarily on weight. Therefore, we removed this statement.		
	4) “Most” and “many” are ambiguous in this context, violating manner and quantity. For example, roughly 30% of overweight people have Type 2 Diabetes. Thus, it is true that “most” overweight people do not develop type 2 diabetes, but being overweight still indicates substantial risk. Removing this statement was a more minimal revision than adding sufficient detail to satisfy maxim of quantity.		
	The overall effect of emphasizing other factors, claiming that it is unfortunate they are ignored, and saying that most overweight people do not develop diabetes, is to falsely suggest that weight is not an important risk factor for diabetes.		
3	**Myth:** Eating too much sugar causes diabetes.		
	**Question:** Does eating too much sugar cause diabetes?		
	**ADA’s explanation:** The answer is not so simple. Type 1 diabetes is caused by genetics and unknown factors that trigger the onset of the disease; type 2 diabetes is caused by genetics and lifestyle factors.^1^		**Revised Explanation:** Research has shown that drinking sugary drinks is linked to type 2 diabetes. The American Diabetes Association recommends that people should avoid intake of sugar-sweetened beverages to help prevent diabetes. Sugar-sweetened beverages include beverages like: regular soda, fruit punch, fruit drinks, energy drinks, sports drinks, sweet tea, [and] other sugary drinks.
	Being overweight does increase your risk for developing type 2 diabetes, and a diet high in calories from any source contributes to weight gain.^2^ Research has shown that drinking sugary drinks is linked to type 2 diabetes.^3^		
			Just one 12-ounce can of regular soda has about 150 calories and 40 grams of carbohydrate. This is the same amount of carbohydrate in 10 teaspoons of sugar! One cup of fruit punch and other sugary fruit drinks have about 100 calories (or more) and 30 grams of carbohydrate.
	The American Diabetes Association recommends that people should avoid intake of sugar-sweetened beverages to help prevent diabetes. Sugar-sweetened beverages include beverages like: regular soda, fruit punch, fruit drinks, energy drinks, sports drinks, sweet tea, [and] other sugary drinks.		
			These will raise blood glucose and can provide several hundred calories in just one serving! Being overweight does increase your risk for developing type 2 diabetes, and a diet high in calories from any source contributes to weight gain.
	These will raise blood glucose and can provide several hundred calories in just one serving!		
	See for yourself: Just one 12-ounce can of regular soda has about 150 calories and 40 grams of carbohydrate. This is the same amount of carbohydrate in 10 teaspoons of sugar! One cup of fruit punch and other sugary fruit drinks have about 100 calories (or more) and 30 grams of carbohydrate.		
	**Analysis**		
	1) These statements violate the principles of relevance, quantity and manner. They violate relevance by focusing primarily on issues other than sugar intake. They violate quantity by providing extraneous information: Additional irrelevant information is given about type 1 diabetes and about genetic risk factors. Finally, the vague use of “lifestyle factors” violates manner by obscuring the fact that sugar intake constitutes a lifestyle factor. These points bear indirectly on the issue of sugar’s causal role, but too little information is given to make this connection. We removed these irrelevant statements.		
	2) Violates principles of relevance and quantity: By focusing on calories and weight gain, this statement falsely suggests there are no direct effects of sugar on type 2 diabetes risk. We moved this sentence to follow a sentence that explains the caloric impacts of sugar, clarifying its relevance.		
	3) Failure to make this point before other, less relevant points violates the principle of relevance. We moved this statement to the beginning of the explanation.		
	Altogether these violations underplay the role of high sugar diet in Type 2 Diabetes.		
4	**Myth:** People with diabetes should eat special diabetic foods.		
	**Question:** Should people with diabetes eat special diabetic foods?		
	**ADA’s explanation:** A healthy meal plan for people with diabetes is generally the same as a healthy diet for anyone ^1^– low in saturated and trans fat, moderate in salt and sugar, with meals based on lean protein, non-starchy vegetables, whole grains, healthy fats and fruit. Diabetic and "dietetic" foods ^2^ generally offer no special benefit. Most of them still raise blood glucose levels, are usually more expensive and can also have a laxative effect if they contain sugar alcohols. ^3^		**Revised explanation:** Unfortunately, there are some foods marketed as “diabetic” or “dietetic” foods, which are claimed to be especially beneficial for people with diabetes, but in fact, these foods generally offer no special benefit. Most of them still raise blood glucose levels, are usually more expensive and can also have a laxative effect if they contain sugar alcohols. Instead, people with diabetes should strive to maintain a healthy diet. Everyone should have a diet low in saturated and trans fat, moderate in salt and sugar, with meals based on lean protein, non-starchy vegetables, whole grains, healthy fats and fruit.
	**Analysis**		
	1) This sentence violates the principles of quality, quantity and manner. This statement could mislead people to infer that people with diabetes can eat the same diet as people without diabetes, which will further imply that people with diabetes do not need to change their diet. But the important claim is that both people with and without diabetes are advised to maintain a healthy diet. We clarified that people with diabetes and everyone else should follow a healthy diet.		
	2) Introducing “diabetic” and “dietetic” foods without defining these terms violates the principle of manner. Many people may be unaware of foods that are marketed for people with diabetes. We provided a brief definition of “diabetic” and “dietetic” foods.		
	3) The crucial point that diabetic foods not only offer no special benefit but may also make the condition worse was not presented until the end. This violates the principle of relevance. In the revised explanation, this point was made first.		
	Overall, the ADA’s explanation could lead people to believe that people with diabetes do not need to change their diets.		
6	**Myth:** People with diabetes can't eat sweets or chocolate.		
	**Question:** Can people with diabetes eat sweets or chocolate?		
	**ADA’s explanation:** If eaten as part of a healthy meal plan, or combined with exercise, sweets and desserts can be eaten by people with diabetes. They are no more "off limits" to people with diabetes than they are to people without diabetes. ^1^ The key to sweets is to have a very small portion and save them for special occasions ^2^ so you focus your meal on more healthful foods.		**Revised explanation:** The key to sweets is to have a very small portion and save them for special occasions so you focus your meal on more healthful foods. Even people without diabetes should limit sweets. Sweets and desserts should only be eaten as part of a healthy meal plan, or combined with exercise.
	**Analysis**		
	1) This sentence violates the principles of quality, quantity, and manner. Claiming sweets are “no more off limits” can be readily misconstrued to mean that people with diabetes are free to eat sweets frequently and in large amounts, as many people without diabetes do. In the revised explanation, this sentence was disambiguated so that it is clearly also about the recommendations for people without diabetes.		
	2) This violates the maxim of relevance. The crucial point that sweets need to be eaten in very small portions and only on special occasions is delayed until the final sentence. Our revised explanation made this point first.		
	Taken together, the ADA’s explanation could lead people to believe that people with diabetes do not have to limit their intake of sweets.		
8	**Myth:** People with diabetes are more likely to get colds and other illnesses.		
	**Question:** Are people with diabetes more likely to get colds and other illnesses?		
	**ADA’s explanation:** You are no more likely to get a cold or another illness if you have diabetes. However, people with diabetes are advised to get flu shots. This is because any illness can make diabetes more difficult to control, and people with diabetes who do get the flu are more likely than others to go on to develop serious complications.		**Revised explanation:** Although you may not be more likely to get a cold if you have diabetes, people with diabetes who do get the flu, for example, are more likely than others to go on to develop serious complications and any illness can make diabetes more difficult to control. For this reason, people with diabetes are advised to get flu shots.
	**Analysis**		
	1) Focusing solely on the likelihood of contracting an illness without considering the severity or risk of complications from an illness violates the maxims of quality and relevance. We rewrote this sentence to read, “Although you may not be more likely to get a cold if you have diabetes …”, to introduce a contrast between likelihood and severity of illnesses.		
	2) This statement violates the principle of quality, quantity, and manner. Depending on the scope of “another illness,” this statement might readily be false. For example, people with diabetes have an increased risk of getting pneumonia. Given this ambiguity, we kept “cold” and “flu” but deleted “other illnesses” in the revised explanation.		
	Overall, the ADA’s explanation could mislead people to infer that diabetes has little detrimental effect on the immune system.		

Text of ADA’s myths and ADA’s explanations reflects how this content appeared on the ADA website when we originally accessed them: July 18, 2017

## Method

This study was approved by the Institutional Review Board Panel on non-medical human subjects at Stanford University (protocol: IRB-14174). Participants’ consent was obtained on a form presented online as part of the survey procedure.

### Design overview

In this study we (1) analyzed how the communicative violations that drive paltering could have made the ADA’s explanations misleading and (2) revised the ADA’s explanations to avoid or minimize paltering. If our revisions were effective, these edited explanations should no longer have detrimental effects on people’s understanding.

To test this, we compared people’s knowledge of diabetes in six experimental conditions. The six conditions differed in the informational content that was presented before the diabetes knowledge test. This information included either the ADA’s original myths or those myths reframed as questions either alone, or paired with the ADA’s explanations or our revised explanations, resulting in a 2 (framing: myth-framing, question-framing) x 3 (presentation of explanations: no explanation, ADA’s explanation, revised explanation) factorial design.

Powell et al. [12] found that participants’ performance in the rephrased questions condition was identical to performance in a baseline control condition in which participants did not receive any information about diabetes. Thus, in the current study, the questions only condition served as a baseline condition. Further, we expected to replicate the earlier finding that the ADA’s explanations undermined participants’ baseline understanding of diabetes. Finally, in the key novel conditions, we presented edited explanations that avoided paltering. We predicted that these revised explanations should not impair knowledge when presented alongside the felicitous questions, and may even correct misconceptions induced by the infelicitous myths.

### Participants

A sample size of 50 participants per condition was chosen based on the results of Powell et al. [12]. A total of 328 participants living in the United States were recruited from Amazon's Mechanical Turk service. Thirty-two participants were excluded from analyses after failing an attention check question, leaving 296 participants (Mean age = 33.95; 189 women, 107 men) in the final analysis. All participants were paid $0.75 for participation.

Among the people who responded, 14.9% reported that they have been diagnosed with diabetes. Among the non-diabetic participants, 14.6% reported that they are prediabetic and 67.8% reported that they have a family member or someone else who is close that has been diagnosed with diabetes.

Sixty-one percent of the participants were non-Hispanic white, 22.6% Asian American, 9.8% Black or African American, 4.4% Hispanic or Latino, 1.0% Native American and 1.0% other. The median yearly household income was $30,001 to $50,000. Sixty-four percent of the participants in the sample had at least a bachelor’s degree.

### Procedure and materials

Participants were directed to an online Qualtrics survey. After a brief demographic questionnaire, they were asked to read some information from the American Diabetes Association website.

Next, participants were randomly assigned into one of six conditions in a 2 (framing: myth-framing vs. question-framing) X 3 (presentation of explanations: no explanation, ADA explanation, or revised explanation) between-subjects design. A first group of participants was recruited and each participant was randomly assigned to one of the three question-framing conditions. After reflection, we determined that another set of conditions where participants were exposed to the myths was needed to properly align the current study with the prior work of Powell et al. [12]. A second group of participants was recruited and each participant was randomly assigned to one of the three myths-framing conditions. For simplicity, we present these two phases together as a single experiment. We identified 5 explanations with pragmatic violations on the American Diabetes Association website (diabetes.org). For each explanation, we revised the language to avoid paltering, generating 5 revised explanations in total. A summary of the original and the revised explanations are presented in Table 1.

Participants in the myth-framing conditions were told they would read some myths about diabetes and then presented with the 5 relevant “diabetes myths” from the ADA’s website. Participants in the question-framing conditions were told they would read about some common questions that people have about diabetes and were then presented with 5 questions reframed from the myths. Alongside the myths or the rephrased questions, participants either read no explanations, the ADA’s explanations, or our revised explanations.

To measure participants’ diabetes knowledge in each condition, we adopted the ten true-false items from Powell et al. [12] (Table 1). The correct answers to these questions were determined based on information available on ADA websites as documented by Powell et al. [12] and reproduced in supplemental materials. Participants were asked to judge if each statement was true or false on a 4-point scale (“definitely true”, “probably true”, “probably false”, or “definitely false”). The scale allowed us to assess both participants’ knowledge accuracy and their confidence in their answers.

**Table 2:** Diabetes knowledge questions and answer sources (* correct answer is false). Table reproduced from Powell et al. [12].

	*Statements to judge as true or false*	*Reference from the ADA’s websites for the correct answers*
1	Being overweight significantly increases the likelihood that someone will become diabetic.	"Being overweight raises your risk for type 2 diabetes, heart disease and stroke.” [19]
2	Losing weight and improving your diet are among the most important things you can do to prevent and control diabetes.	"Eating well to reach or stay at a healthy weight is one of the most important things you can do to lower your risk for type 2 diabetes." [20]
3	Even losing a modest amount of weight can help prevent or control diabetes.	". . .you don't have to lose a lot of weight to improve your health—even losing 10–15 pounds can make a big difference.” [19]
4	People with diabetes have a compromised immune system and are more likely to have serious infections.	"Studies have shown that the white blood cells in people with both type 1 and type 2 diabetes don’t function as well in attacking foreign bacteria” [21]
		"People with uncontrolled type 1 or 2 diabetes are more susceptible to infections in the first place because of poor immune function." [22]
5	People with diabetes are more likely to suffer serious consequences of a range of diseases including the flu, pneumonia, and periodontal disease.	"People with diabetes are at a higher risk for gum disease and other dental problems. Diabetes may weaken your mouth and body's germ-fighting powers and high blood glucose levels can make gum disease worse." [23]
		"People with diabetes are about three times more likely to die with flu and pneumonia." [24]
6	There is no special diet for people with diabetes, so most can continue eating their usual meals without making substantial changes.*	The recommended diet is ". . .low in saturated and trans fat, moderate in salt and sugar, with meals based on lean protein, non-starchy vegetables, whole grains, healthy fats and fruit." (Fact #4)
7	People with diabetes can include chocolate and other sweet desserts as part of their regular diet just like people without diabetes.*	"The key to sweets is to have a very small portion and save them for special occasions" (Fact #6)
8	Reducing carbohydrate intake is important for controlling and preventing diabetes.	"Counting carbohydrate can help you reach your blood glucose goals and prevent diabetes complications.... A general guideline is to have: 45–60 grams of carbohydrate at each meal; 15–20 grams of carbohydrate servings at each snack" [25]
		"Starchy foods can be part of a healthy meal plan, but portion size is key" (Fact #5)
9	Lifestyle changes can often let people control their diabetes without medication or with reduced medication.	"You can prevent or delay type 2 diabetes by: losing weight, cutting back on calories and saturated fat, increasing your daily physical activity" [26]
10	Many people can prevent or delay type 2 diabetes by making healthy lifestyle choices.	

Participants were also asked to enter “probably false” for an attention check question included in the questionnaire. Those who failed this attention check were excluded from analyses.

## Result

The main goal of this study was to determine whether correcting the pragmatic violations in the ADA’s explanations would mitigate their negative effects on people’s diabetes knowledge.

**Fig 1:**
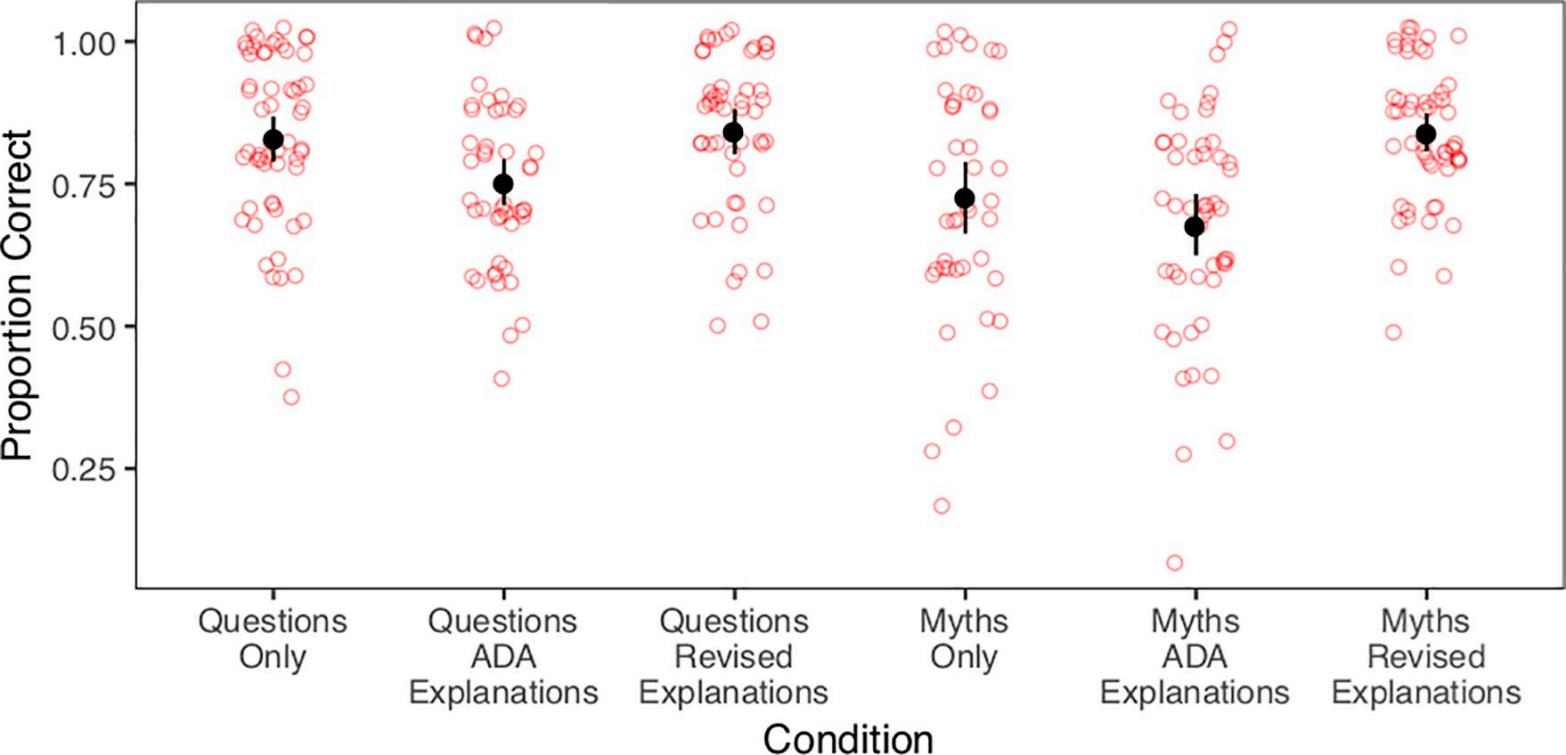
Proportion of correct responses across the 10 items. Red, circular dots show proportion correct for each individual participant (with a small amount of jitter). Black, filled points and bars indicate average proportion correct for each condition, with 95% bootstrapped confidence intervals.

**Fig 2:**
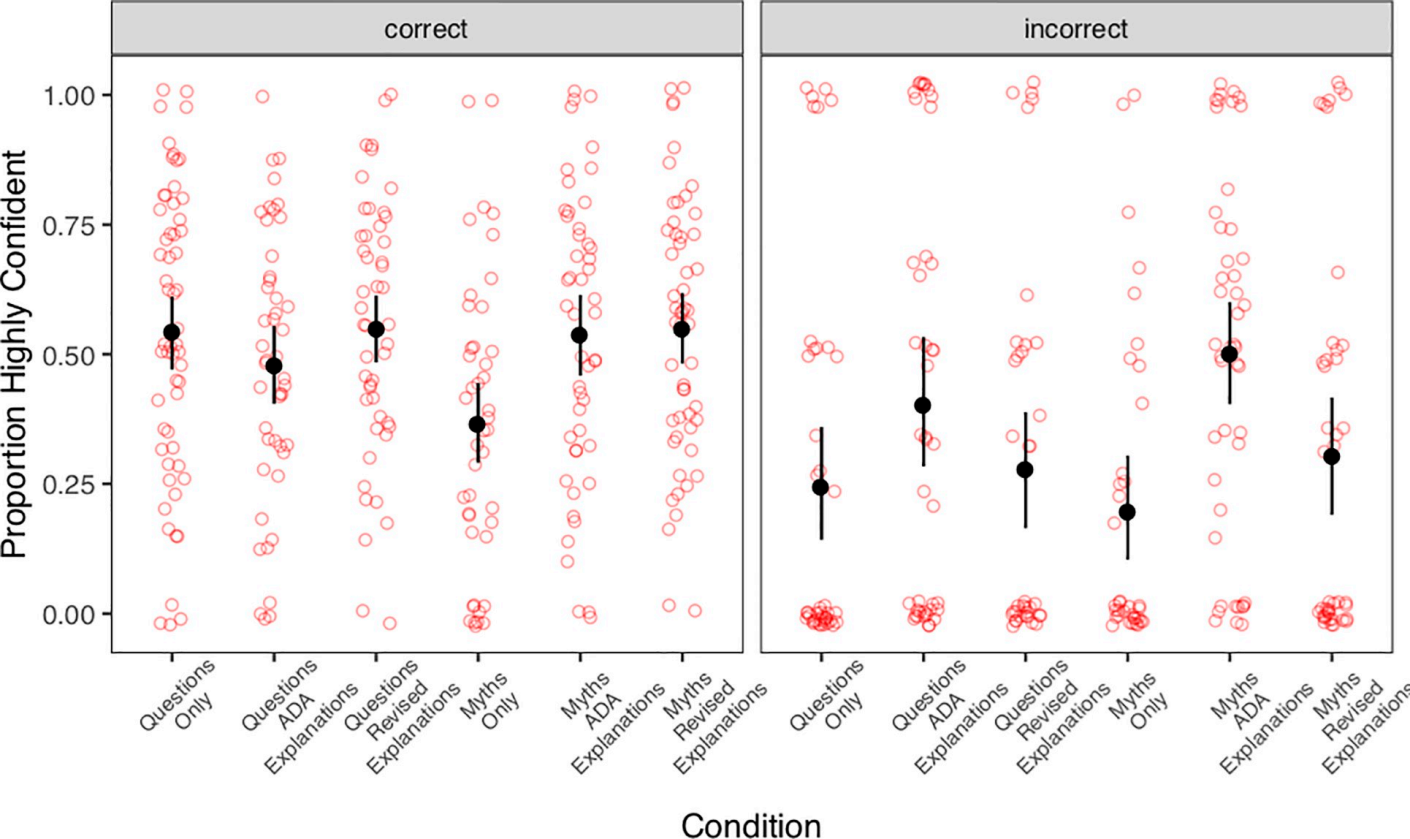
Proportion of highly confident responses for correct (left) and incorrect (right) responses. Red, circular dots show proportion correct for each individual participant (with a small amount of jitter). Black, filled points and bars indicate average proportion correct for each condition, with 95% bootstrapped confidence intervals.

Recall, participants were asked to judge if each statement was true or false on a 4-point scale (“definitely true”, “probably true”, “probably false”, or “definitely false”). Participants’ responses were re-coded as a binary accuracy measure, indicating correct and incorrect responses. The proportion of correct responses across conditions is shown in Fig 1. We also re-coded participants’ responses as either high or low confidence for both correct and incorrect answers (Fig 2).

We report Bayesian hierarchical regression analyses conducted using the BRMS R package (v2.2). The BRMS package implements Bayesian analyses using the probabilistic programming language Stan [27]. This approach allows us to model all of the data by using responses for each individual question rather than aggregating values for each participants, and to apply models that are consistent with the data generating process (e.g., applying logistic regression for binary responses). We assume a weakly-informative prior, Normal(0,1), for betas in each model, as suggested by Gelman and colleagues [28].

1. The Accuracy of Diabetes Knowledge

First, we examine how the presentation of information affected participants’ accuracy on the diabetes knowledge test. We examined condition differences by predicting accuracy (correct or incorrect) on each question from variables representing the myths and explanation type factors and their interactions and with random intercepts by item and by participant. This hierarchical logistic regression model can be expressed in the common mixed-effects formula syntax, as:

accuracy∼myths_cond∙exp_cond+(1|item)+(1|participant)

We first examined whether the primary findings of Powell et al. [12] were replicated in our new study. In prior work, Powell et al. [12] found that the infelicitous “myths” negatively impacted participants’ diabetes knowledge, but that diabetes knowledge was preserved when the myths were rephrased as questions. Replicating these findings, we found that diabetes knowledge was reduced by the myths phrasing relative to the rephrasing as questions, as the myths factor coefficient was credibly negative, B = -0.49, 95% Credible Interval (*CI*_*95*_) = [-0.97, -0.04]. In addition, Powell et al. [12] found that, rather than improving diabetes knowledge, the ADA’s explanations actually reduced diabetes knowledge. We replicated this finding again in our current study: Participants who read the ADA’s explanations scored worse on the diabetes knowledge test than participants who read no explanations at all, B = -.61, *CI*_*95*_ = [-0.15, -1.08].

Having replicated these findings, we then examined the impact of our revised explanations, which better respected Gricean communicative norms, on participants’ diabetes knowledge. As predicted, we found that our revised explanations improved participants’ accuracy on the diabetes knowledge test relative to participants who saw the ADA’s explanations, B = .74, *CI*_*95*_ = [0.24, 1.22].

To further examine differences among the conditions, we performed additional comparisons among the obtained condition coefficients. For each comparison, we report the mean difference between the estimated condition coefficients (B_diff_) with 95% credible intervals. First, we examined whether our revisions to the explanations actually rendered them informative, as opposed to merely not misleading. First, we examined whether the revised explanations were able to correct the misleading effects of the ADA’s infelicitous “myths” statements. Compared to participants who read the myths alone, performance on the diabetes knowledge test was superior among participants who read the myths paired with the revised facts, B_diff_ = .837, *CI*_*95*_[1.365, .314]. Powell et al. [12] found that participants who read the myths rephrased as questions performed similarly on the diabetes knowledge test as participants in a baseline condition who read no information. Comparing participants who read the questions versus those who read the questions paired with our revised explanations, we did not see a significant improvement in diabetes knowledge B_diff_ = .181, *CI*_*95*_ [-0.308, .675]. Thus, our revisions are informative enough to clarify or undo the damage done by the infelicitous myths, but they do not educate participants beyond baseline. This may be due to the relatively basic level of diabetes knowledge being addressed and assessed in this study.

To summarize, both the presentation of myths and the ADA’s explanations reduced participants’ knowledge of the basic diabetes facts. In contrast, our findings indicate that when the ADA’s explanations were revised to eliminate the pragmatic violations, they restored participants’ knowledge of diabetes.

2. Confidence in Correct and Incorrect Answers

Next, we considered how these materials influenced people’s confidence in their answers. Ideally, educational materials should increase people’s confidence in their correct responses and reduce their confidence in incorrect responses. Fig 2 shows participants’ average confidence across items for correct and incorrect responses.

### Confidence in correct answers

In prior work we found that the ADA myths undermined people’s confidence in their correct responses, but pairing the ADA’s explanations with the myths helped restore this confidence [12]. To examine whether this effect was replicated, participants’ confidence for correct answers were submitted to a hierarchical logistic regression analysis, predicting degree of confidence (binary, high or low) from variables representing condition and with random intercepts for item and participants. Expressed in mixed-effects formula syntax, the model was:

confidence∼condition+(1|item)+(1|participant)

First we assessed whether the infelicitous myths reduced confidence in correct responses. Compared to the questions-only condition, participants’ confidence in correct answers was again reduced in the myths-only condition, B = -0.90, 95% CI = [-1.44, -0.37]. Then, we examined how the explanations affected confidence for correct responses. Further replicating prior findings, the ADA’s facts did restore confidence in correct answers relative to the myths-only condition, B_diff_ = .835, 95% CI = [.258, 1.432]. Moreover, our revised explanations restored confidence in correct responses to a similar degree, B_diff_ = .928, 95% CI = [.375, 1.495].

### Confidence in incorrect answers

Finally, we performed the same analysis for participants’ confidence for incorrect answers. First we assessed whether the infelicitous myths increased confidence in incorrect responses. Compared with the questions-only condition, the myths and ADA’s facts increased participants’ confidence in their incorrect answers, B = 1.29, 95% CI = [0.59, 2.00]). A similar pattern was observed for the questions and ADA facts condition, although the posterior distribution of this coefficient credibly includes zero, B = 0.68, 95% CI = [-0.08, 1.44].

The presence of ADA’s explanations inflated confidence in incorrect responses, indicating that these explanations generated additional confusion about basic diabetes knowledge. In contrast, our revised explanations without pragmatic violations not only protected confidence in correct responses, but did so without also inflating confidence in incorrect responses.

## Discussion

In this study, we identified and analyzed pragmatic violations on a prominent health website that we argue led to inadvertent paltering, rendering their explanations misleading rather than informative. In prior work, we identified how information on the American Diabetes Association’s “diabetes myths” page mislead health consumers through the infelicitous labeling of “myths.” Here, we turned to the more detailed explanations accompanying the “myths” on this page, and determined that they violated, at one point or another, each of the Gricean cooperative principles of quality, quantity, manner, and relevance. By revising these explanations to better adhere to the pragmatic maxims while preserving their content, we were able to minimize the materials’ misleading effects. These revised explanations were no longer misleading by themselves and, more importantly, were able to counter the confusion generated by the ADA’s infelicitous myths. To take a striking example, recall that one of these “myths” was that “People with diabetes are more likely to get colds and other illnesses.” Among participants who read the material as presented on the ADA’s website, containing both this myth and its pragmatically flawed explanation, only 41% correctly indicated that “People with diabetes have a compromised immune system and are more likely to have serious infections.” However, when the myths were paired with our revised explanation, 76% of the participants answered correctly. Thus, by correcting the Gricean violations in the original explanations, we were able to transform them from misleading to educational.

When people seek health information from reputable, expert sources, they expect those sources not only to convey accurate information, but also to guide and empower them to make positive health decisions. To be successful, material must be conveyed to readers in ways that honor the Gricean maxims. This is true for all of the maxims, but the maxim of quality may have special force when the speaker is an expert. This maxim sets the expectation that communications are not just truthful, but that the speaker has evidence or justification for what they say. People assume that experts draw on a large body of knowledge and evidence to back their assertions. This leads readers to have more credence in the information they receive from experts and to feel greater confidence to act on that information.

Inadvertent pragmatic violations may be more likely to occur when speakers have conversational goals beyond conveying truth, such as avoiding stigma, empowering patients, fostering inclusivity, and so forth (e.g., [29,30,31]). In this light, we suspect that ADA had the additional goals of not blaming or stigmatizing people who have diabetes and to avoid making the recommended lifestyle changes appear too difficult to achieve. It is possible that these goals have led the ADA to understate the role of lifestyle choices in diabetes and to underplay the control diabetic people can achieve over their own health.

We suspect that dramatic instances of inadvertent paltering like the ADA’s myths page are rare, but less egregious violations may be more common in health communications. There is evidence that the tension between goals of conveying information and avoiding upsetting patients is widespread in the medical profession. For example, although health professionals believe that people should be informed about their prognosis, many of them choose to withhold information to avoid depressing their patients [32]. Fallowfield, Jenkins, and Beveridge [33] presented an example of a doctor attempting to prepare a patient with lung cancer for a transition from curative to palliative care. The doctor told the patient “there are signs that things are progressing so we do not think that you should have anymore chemotherapy”. The patient believed that the doctor was saying that “things,” meaning the treatment, had progressed so well that there is no need to continue. What the doctor actually meant was that “things,” meaning the cancer, were progressing so aggressively that further treatment would not be effective. By speaking euphemistically rather than straightforwardly, the doctor inadvertently paltered and misled the patient.

Striving to avoid upsetting people can come at the cost of accurately conveying information. People who visit the ADA’s website are most likely to be primarily seeking advice for how they can avoid becoming diabetic or manage their diabetes. Underplaying the degree to which they can control their health may lead them to infer that there is little they can do or that there is no strong need for them to take action. Therefore, educators and science or health communicators should be conscious of their communicative goals. In some cases, it may be better to be more open and straightforward about these additional goals, rather than to subtly work to accomplish them unnoticed (e.g., see [34]). If these subtle machinations are not deftly realized, there is a considerable danger that unintentional paltering can result, and the public can be misled.

These concerns are all the more pressing when considered in light of the overwhelming evidence demonstrating the difficulties inherent in correcting misinformation once fallacious beliefs have taken hold (for a review see [35]). Because it is difficult to undo the damage of misinformation [36,37,38,39,40], health organizations have a responsibility to “do no harm,” and to be clear and considerate in their communications. Of course, this isn’t to say that correcting misconceptions is impossible; for instance, recent work examining educational interventions aimed at countering vaccine skepticism have shown improvements in participants’ attitudes toward vaccines [41]. One key to addressing misconceptions may be developing interventions that acknowledge and target the wider beliefs driving misconceptions [12]. Where misconceptions exist among the public, it is the responsibility of health organizations to combat them. Websites and social media posts are now an essential means for informing the public, so health organizations should work to ensure that the information presented on these forums is as clear and accurate as possible (cf. [42]).

Paltering may be seen as one member of a family of misleading communicative practices, among them bullshit [43], pandering [9], and white lies (e.g., [44]). Bullshitters attempt to persuade others without regard for truthfulness [43]. Likewise, panderers flatter without concern for the truth [9]. Those who tell white lies inflate the positives or twist the truth to spare others’ feelings. On our analysis, paltering results from the violation of communicative principles. That same kind of analysis might shed light on these other forms of deception. As a step in this direction, Yoon et al. [44] analyze white lies by assuming speakers balance two goals: truth and the feelings of the listener. Depending on the true state of affairs and the relative weights of these goals, speakers might choose to lie entirely (“You did an excellent job!” when it was a mediocre job) or to be truthful but not maximally informative (“Not bad!” when it was a mediocre job) which is more like paltering. It could be interesting to extend this line of work by considering the ways in which violations of Gricean norms underlie white lies as they do paltering.

To conclude, prior work on paltering has examined a number of different techniques by which the truth can be used to mislead, including misplaced emphasis, omissions, and vagueness. We argued that these diverse techniques are unified as violations of Gricean communicative norms. These same kinds of violations can occur without an explicit intent to deceive which can result in unintentional paltering. Intentional palterers abuse communicative norms to mislead their listeners with true statements. As our findings illustrate, the neglect of these norms can be just as detrimental to communication as their abuse.
